# Cannabis in Chinese Medicine: Are Some Traditional Indications Referenced in Ancient Literature Related to Cannabinoids?

**DOI:** 10.3389/fphar.2017.00108

**Published:** 2017-03-10

**Authors:** E. Joseph Brand, Zhongzhen Zhao

**Affiliations:** School of Chinese Medicine, Hong Kong Baptist UniversityHong Kong, Hong Kong

**Keywords:** Cannabis, Chinese medicine, historical changes, bencao, cannabidiol

## Abstract

*Cannabis sativa* L. (Cannabaceae) has a long history of utilization as a fiber and seed crop in China, and its achenes (“seeds”) as well as other plant parts have been recorded in Chinese medical texts for nearly 2000 years. While the primary applications of cannabis in Chinese medicine center around the use of the achenes, ancient indications for the female inflorescence, and other plant parts include conditions such as pain and mental illness that are the subject of current research into cannabinoids such as cannabidiol (CBD) and Δ^9^-tetrahydrocannabinol (THC). However, little previous research has been conducted to analyze the Chinese medical literature in light of recent advances in the pharmacology and taxonomy of cannabis, and most of the relevant Chinese historical records have not yet been translated into Western languages to facilitate textual research. Furthermore, many key questions remain unresolved in the Chinese literature, including how various traditional drug names precisely correspond to different plant parts, as well as the implications of long-term selection for fiber-rich cultivars on the medical applications of cannabis in Chinese medicine. In this article, prominent historical applications of cannabis in Chinese medicine are chronologically reviewed, and indications found in ancient Chinese literature that may relate to cannabinoids such as CBD and Δ^9^-THC are investigated.

## Introduction

*Cannabis sativa* L. has been cultivated in China for millennia for use as a fiber, food, and medicine. References to cannabis are found throughout classical Chinese literature, including in many famous works of philosophy, poetry, agriculture, and medicine. Fiber-rich biotypes of cannabis (hemp) were extensively used in ancient China for clothing and the production of paper, rope, and fishing nets (Dai, 1989), and the achenes (“seeds”) of cannabis have been continuously used in Chinese medicine for at least 1800 years. Today, China is regarded as one of the world's ancient epicenters of hemp cultivation, resulting in a diverse germplasm with genetically distinct regional varieties of fiber-rich hemp that are adapted to local environmental conditions throughout the country (Gao et al., 2014; Zhang et al., 2014).

The prominence of hemp in ancient Chinese culture can be seen by its occurrence in classical literature from the Warring States Period (475–221 BC), including philosophical works by Confucius, Mencius, Xunzi, Zhuangzi, and Mozi, as well as the *Classic of Poetry* (*Shi Jing*; Sun, 2016). By the first to second century AD, the ancient *Shuowen* dictionary (*Shuo Wen Jie Zi*) featured multiple Chinese characters that illustrate knowledge of the dioecious nature of cannabis and discriminate based on gender (Liu, 1999).

In the sixth century AD, the agricultural text *Essential Techniques for the Welfare of the People* (*Qi Min Yao Shu*) described techniques for the cultivation of hemp in great detail, and its monograph on cannabis cultivation features one of the first textually documented applications of fertilizer in the history of Chinese agriculture (Shi, 1957). This text also demonstrates the knowledge that removal of male plants at the initiation of flowering will result in a lack of seeds; however, the text focuses exclusively on cultivation and harvesting practices to maximize the production of seeds and the quality of fiber and does not reference the deliberate production of seedless cannabis (Shi, 1957).

**Figure 1:**
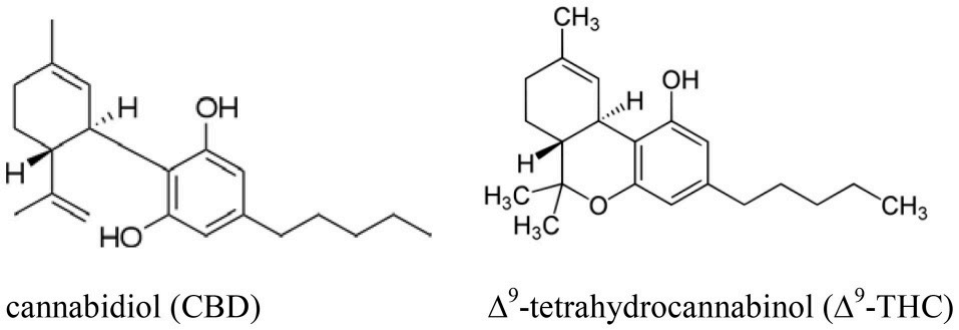
**The chemical structures of cannabidiol (CBD, left) and Δ9-tetrahydrocannabinol (Δ9-THC, right)**.

It is notable that most classical Chinese references focus on the use of cannabis for its seeds and fiber, with few, if any, explicit references to drug effects seen outside of the medical literature. Although early Chinese medical literature suggests that both drug and fiber biotypes of cannabis were known in ancient times, more research is needed to clarify the implications of these different biotypes in medical applications. Additionally, further research is needed to probe whether the medical applications of cannabis in ancient Chinese literature may relate to non-psychoactive cannabinoids such as cannabidiol (CBD), which may have been present in ancient fiber biotypes as well as drug biotypes (see Figure 1).

## Cannabis in Chinese medicine

Cannabis has been continually documented in Chinese medicine for ~1800 years. In the modern era, its achenes (commonly referred to as “seeds” and known in TCM as *huomaren* 火麻仁) are frequently used as a moistening laxative and are official in the Chinese Pharmacopeia (CP, 2015). All parts of the cannabis plant have been recorded in historical Chinese medical texts, including the achene (seed), female inflorescence, leaf, and root, as well as the cortex of the stalk and the water used to process the stalk into fiber. However, only the achenes (seeds) are currently used in clinical practice (Brand and Wiseman, 2008).

In contrast to the prominent use of the achenes in Chinese medicine, many applications of cannabis in early Western medicine focused on preparations made from the female flowering tops of drug varieties of cannabis, which were featured in early Western pharmacopeia texts from the nineteenth to twentieth century (Wood, 1918). In the modern era, the investigation of cannabis for medical purposes in the West has continued to primarily focus on cannabinoids, resulting in prescription medicines such as the botanically derived drug “Sativex” by GW Pharmaceuticals (a mixture of Δ^9^-THC and cannabidiol in an oromucosal spray that is sold by prescription in 15 countries, including the UK, Germany, Italy, Canada, Australia, and Spain; Russo et al., 2007).

The notable contrast between the medical applications of cannabis in traditional Chinese medicine and Western medicine has been poorly explored in current ethnopharmacological literature. Despite the fact that cannabis preparations have been extensively and consistently documented in Chinese *bencao* (materia medica) texts for ~1800 years, no English-language publications have systematically assessed the medicinal indications of cannabis in the Chinese *bencao* literature and historical changes in the plant parts used. Few reliable translations of Chinese monographs on cannabis from traditional *bencao* texts exist, which has led to significant gaps in the Western understanding about how cannabis was used in Chinese medicine.

Additionally, many problems related to cannabis in TCM remain unresolved in the contemporary Chinese literature. Modern Chinese journal articles as well as historical authors have attempted to clarify the complicated nomenclature of the female inflorescence in *bencao* literature (Liu and Shang, 1992; Liu, 1999; Liu et al., 2009; Wei et al., 2010), and monographs in modern TCM texts detail different plant parts and their use across a range of historical texts (Editorial Committee, 1977; Cui and Ran, 1993). However, a number of modern and historical Chinese sources contradict each other in terms of which plant parts correspond to certain traditional drug names such as *mafen* (麻蕡), *mahua* (麻花), and *mabo* (麻勃), complicating the interpretation of their medical actions.

As the difference between drug and fiber varieties of cannabis is largely determined by genetics, the historical and geographic prevalence of different biotypes of cannabis in China likely influenced its applications in Chinese medicine. However, this crucial question has received only limited attention in the Chinese literature. Furthermore, most Chinese publications that have attempted to address the topic of speciation as it relates to the historical application of cannabis in Chinese medicine utilize a relatively simplistic taxonomic model that does not take recent scientific advances into account (Liu and Shang, 1992; Wei et al., 2010).

## Biotypes of cannabis in China

The complicated taxonomic history of cannabis has been previously summarized in numerous publications (Schultes et al., 1974; Small and Cronquist, 1976; Hillig and Mahlberg, 2004). Cannabis is often described as a monotypic genus with wide morphological and chemical variation, and the *Flora of China* and the *Chinese Pharmacopeia* adopt the monotypic classification of Chinese cannabis as *Cannabis sativa* L. (Chen and Gilbert, 2006; CP, 2015). By contrast, many of the Chinese publications that have investigated historical questions related to the speciation of cannabis in Chinese medicine across different dynastic periods have adopted a polytypic approach to nomenclature that primarily differentiates the genus into two species based on chemotype, with varieties focused on fiber and seed production described as *C. sativa* L. and drug varieties described as *C. indica* Lamarck (Liu and Shang, 1992; Liu, 1999).

Cannabis is a classic example of taxonomic debates related to “lumping vs. splitting” (i.e., whether the genus should be considered as monotypic or polytypic) as well as morphological vs. chemotype distinctions. Advances in DNA research have added further complexity to the picture, and terms such as “broad leaflet hemp” (BLH) vs. “broad leaflet drug” (BLD) and “narrow leaflet hemp” (NLH) vs. “narrow leaflet drug” (NLD) have recently been used to describe cannabis varieties based on a combination of morphology and chemotype (Piluzza et al., 2013).

**Figure 2:**
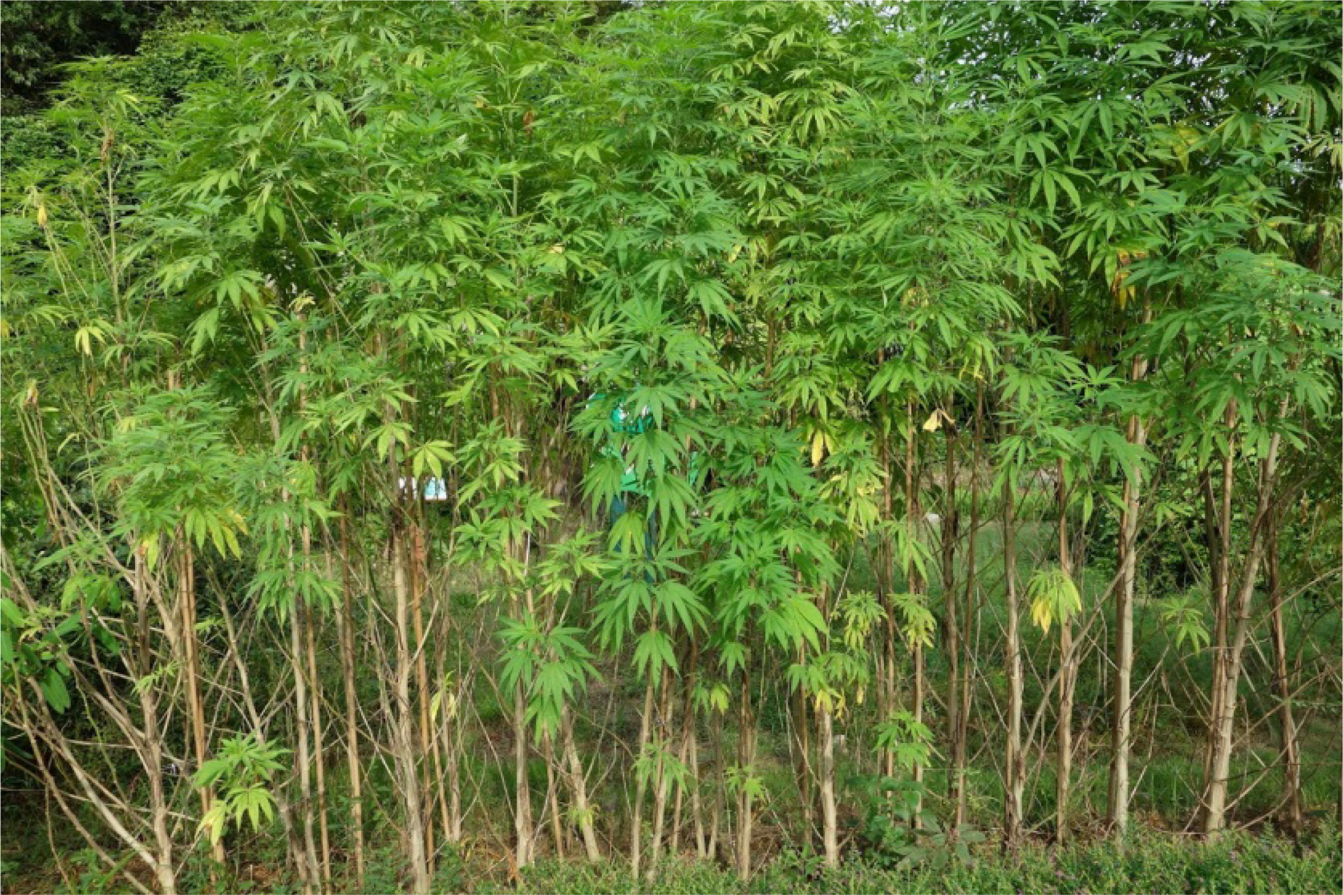
**Broad-leaflet hemp in Guangxi province, China**.

The complex debate about cannabis taxonomy initially developed after Lamarck proposed the name *C. indica* in 1785 to describe psychoactive Indian cannabis in contradistinction to Linnaeus' description of non-psychoactive European hemp (Hillig and Mahlberg, 2004), which was regarded as *C. sativa* L. While Lamarck's original type specimen of *C. indica* reflected a narrow leaflet drug (NLD) variety, Schultes later applied the name *C. indica* to refer to broad leaflet drug (BLD) varieties from Afghanistan that shared the characteristic of psychoactivity but differed in morphology (Clarke and Merlin, 2013). In contrast to European hemp, which is considered as representative of a narrow leaflet hemp biotype (NLH), most landraces of Chinese cannabis are characterized as a broad leaflet hemp (BLH) biotype. (Russo et al., 2008) (see Figure 2). Chinese hemp has strong fiber and is generally not psychoactive, but DNA and chemotype distinctions suggest that Chinese hemp is more closely linked genetically to *C. sativa* subsp. *indica* [ = *C. indica* Lamarck] than to European hemp (*C. sativa* subs. *sativa* [ = *C. sativa* L.]) (Hillig and Mahlberg, 2004; Hillig, 2005). Accordingly, Chinese hemp expresses the B_T_ alleles necessary for the biosynthesis of THC more prominently than European hemp, even though long-term selection for fiber has led Chinese hemp to produce only low levels of THC (Clarke and Merlin, 2013). This suggests that drug and fiber biotypes of cannabis in China may have shared a common ancestor and diverged through human selection, but the precise timeline of fiber hemp's increasing dominance in the Chinese gene pool remains unclear.

Recent archeological evidence from a 2700 year old tomb discovered in the Yanghai region of China's Xinjiang province suggests that drug biotypes of cannabis were known to the ancient inhabitants of the region (Jiang et al., 2006), and genetic testing has shown that the 2700 year old cannabis specimens from the tomb maintain some similarities to feral cannabis that remains in the surrounding region today (Mukherjee et al., 2008). However, the Yanghai tomb housed a body of Caucasian ancestry (Russo et al., 2008), and the region was located well outside of the boundary of early Chinese cultural influence. In more central Chinese regions, archeological artifacts provide abundant evidence of hemp fiber but little evidence of drug cannabis, suggesting that the historical divergence of fiber and drug biotypes occurred early in Chinese history (Clarke and Merlin, 2013).

**Figure 3:**
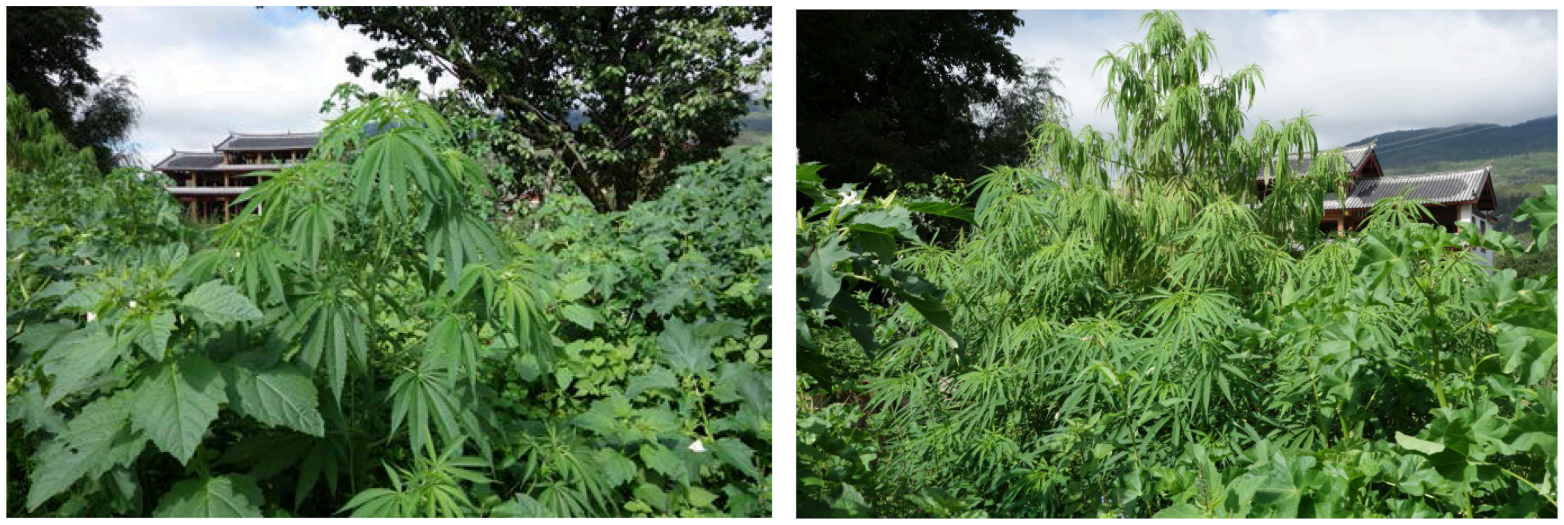
**Feral cannabis in Yunnan province, China**.

Today, cannabis landraces throughout most regions of China reflect fiber biotypes, and some provincial standards have followed international trends by defining fiber biotypes for cultivation as varieties containing <0.3% Δ^9^-THC by weight (Lu et al., 2007). In modern China, intermediate and drug biotypes have primarily been reported in isolated regions in the northwest province of Xinjiang and the southwest province of Yunnan (Zhan et al., 1994; Hu et al., 2015) (see Figure 3). Drug use of cannabis in Xinjiang province was recorded along the Silk Road from the Qing Dynasty (1644–1911 AD) to the twentieth century, and was described in a report by the Russian explorer Shoqan Walikhanov in 1858 (Ali et al., 2004; Zhou, 2015); however, there is little evidence outside of *bencao* literature that suggests that drug cannabis was known or used in other parts of ancient China. Furthermore, according to texts that focus on the history of drug prohibition in China, there is little evidence that cannabis was either abused or prohibited in China prior to the first documented seizures of imported cannabis products in Xinjiang in 1936 (Ali et al., 2004; Qi and Hu, 2004). This stands in notable contrast to other drugs that have a well-documented history of regulation, medical use, abuse, and prohibition in China, such as opium and deliriant drugs derived from tropane alkaloid-containing plants such as henbane (*Hyoscyamus niger* L.) and datura (*Datura* spp.; Li, 1999; Qi and Hu, 2004).

On the whole, the abundance of references to hemp and the paucity of references to drug cannabis in early Chinese history suggest that fiber and drug varieties had diverged by ancient times. As cannabis is wind-pollinated and its biotype distinctions are genetically determined, the long-term and abundant cultivation of fiber-rich biotypes in China likely supplanted or diluted any drug biotypes that were once present. However, the timeline of this process has been poorly elucidated, and *bencao* literature suggests that drug effects of cannabis were recognized in Chinese medicine from ancient times up through Ming dynasty texts written in the sixteenth century AD. This curious anomaly suggests that the evolution of Chinese cannabis biotypes may have taken place gradually, and merits further investigation to determine if *bencao* literature can help to clarify when fiber and drug biotypes diverged in ancient China, and the implications of such a divergence on its medical applications.

## Materials and methods

Pre-modern Chinese materia medica texts, known as *bencao*, were systematically reviewed to investigate the historical applications of different parts of the cannabis plant. In particular, records related to plant parts such as the flowers and leaves were comprehensively investigated for applications that may relate to cannabinoids such as CBD and Δ^9^-THC; such records may also help to clarify the evolution of fiber vs. drug biotypes of cannabis in ancient China.

Representative *bencao* texts were selected for analysis, including influential *bencao* texts from different dynastic periods, thematic *bencao* texts dedicated to specialized topics, and regional *bencao* texts dedicated to specific geographic regions (Zhao and Chen, 2014). Additionally, modern Chinese materia medica compilations as well as texts focused on ethnic minority traditions in China were reviewed. The selected texts were organized chronologically by dynasty, and monographs on cannabis from *bencao* texts representing different historical periods were reviewed. The sources were analyzed based on the plant parts that were described, as well as the nature, flavor, actions and indications of the various cannabis materials within.

A diverse range of properties and indications have been ascribed to various parts of the cannabis plant over the centuries, and space limitations preclude a comprehensive translation of all the *bencao* records related to cannabis. Thus, special attention was given to tracing the historical development of applications related to seizures, pain, and mental effects or mental illness, as these conditions have been the subject of extensive research in the context of cannabinoids such as CBD and Δ^9^-THC (Mechoulam et al., 2002; Devinsky et al., 2014). As cannabinoids are primarily concentrated in the female flowering tops and leaves rather than the achenes, cortex, and roots, these plant parts were emphasized in this review.

## Selection of texts and textual editions

Authentic editions of over 800 historical *bencao* texts are collected together in a set known as the *Complete Ben Cao* (本草全書 *Ben Cao Quan Shu*); a set of this compilation stored at Hong Kong Baptist University was used as a primary reference in this study. In particular, five influential texts from different dynasties are recognized as milestones in *bencao* literature (Zhao and Chen, 2014); these texts were extensively reviewed and are detailed in Table 1. Multiple textual editions were reviewed for each of these *bencao* texts to ensure accuracy and textual integrity.

**Table 1:** **Influential ***bencao*** from different dynastic periods**.

*The Divine Farmer's Classic of Materia Medica* (神農本草經 *Shen Nong Ben Cao Jing*) (Anonymous, Eastern Han Dynasty, 25–220 AD)
*Collection of Commentaries on the Classic of the Materia Medica* (本草經集注 *Ben Cao Jing Ji Zhu*) (Tao Hongjing, early sixth century AD)
*Newly Revised Materia Medica* (新修本草 *Xin Xiu Ben Cao*) (commissioned by the Tang Dynasty government, 659 AD)
*Materia Medica Arranged According to Pattern* (證類本草 *Zheng Lei Ben Cao*) (Tang Shenwei, 1108 AD)
*Compendium of Materia Medica* (本草綱目 *Ben Cao Gang Mu*) (Li Shizhen, 1593 AD)

**Figure 4:**
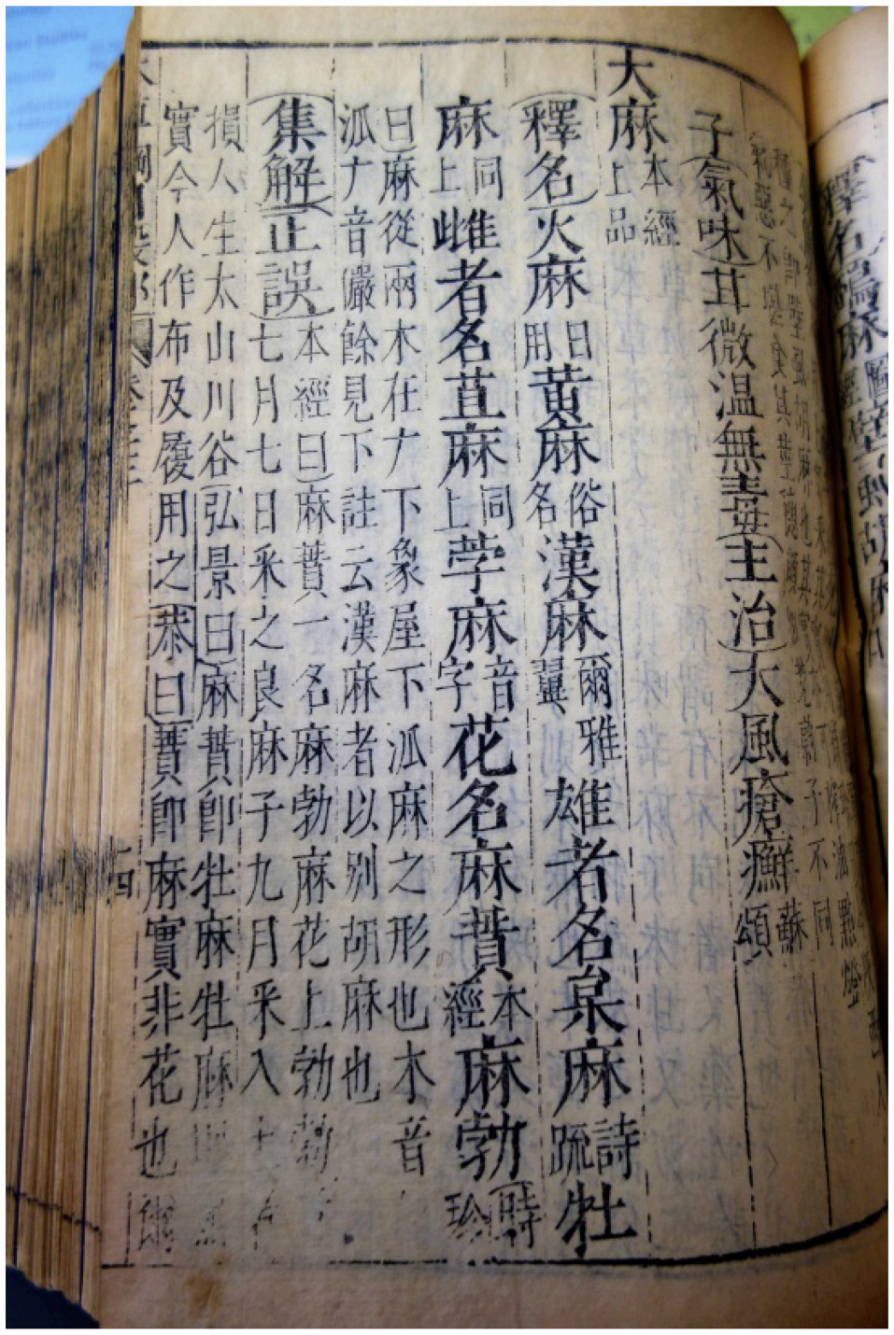
**Entry on cannabis in the Compendium of Materia Medica**.

For example, the longest and most detailed monograph on cannabis in the *bencao* literature is found in the sixteenth century *Compendium of Materia Medica* (*Ben Cao Gang Mu*) by Li Shizhen, which is widely regarded as the pinnacle achievement of *bencao* literature. For the review of this text, three authoritative editions were selected to ensure textual accuracy. The editions reviewed included an original copy of the 1603 AD “Shiquge (石渠閣)” print run of the *Compendium of Materia Medica* from the collection of Sir Hans Sloane at the British Library (see Figure 4), as well as a modern reproduction of the original “Jinling (金陵)” edition of the *Compendium of Materia Medica* (Li, 1999), and an authoritative modern annotated edition known as *Compendium of Materia Medica Research* (Liu et al., 2009).

In addition to these five representative *bencao* texts of different dynasties, records related to cannabis were reviewed from 84 additional pre-modern *bencao* texts dating from the twelfth century to the late nineteenth century. The sources included 6 *bencao* texts from the Song Dynasty (960–1279 AD), 4 texts from the Yuan Dynasty (1271–1368 AD), 32 texts from the Ming Dynasty (1368–1644 AD), and 47 texts from the Qing Dynasty (1644–1911 AD).

Most historical *bencao* texts that were written prior to the invention of printing in the Song Dynasty are no longer intact, but their content has been preserved in printed texts such as the *Materia Medica Arranged According to Pattern* (證類本草 *Zheng Lei Ben Cao*) from 1108 AD. Accordingly, the *Materia Medica Arranged According to Pattern*, which cited 25 previous historical sources in its discussion on cannabis, was selected as a representative source for pre-Song Dynasty content (Tang, 1999). An additional early source of records related to cannabis from the *Treatise of the Five Viscera* (五臟倫 *Wu Zang Lun*), a manuscript discovered in an archeological excavation at Dunhuang that is thought to date from the Tang Dynasty (618–907 AD), was also reviewed.

Additionally, four thematic *bencao* were reviewed to investigate records related to specific geographic regions and imported medicinals. These texts include the tenth century *Materia Medica from Overseas* (海藥本草 *Hai Yao Ben Cao*), the thirteenth century *Materia Medica from Steep Mountainsides* (履巉巖本草 *Lu Chan Yan Ben Cao*), the fifteenth century *Yunnan Materia Medica* (滇南本草 *Dian Nan Ben Cao*), and the nineteenth century *Illustrated Reference of Botanical Nomenclature* (植物名實圖考 *Zhi Wu Ming Shi Tu Kao*).

Finally, monographs on cannabis from a variety of modern materia medica compendiums were reviewed. In addition to each edition of the Chinese Pharmacopeia from 1977 to 2015, several twentieth century compendiums of materia medica were reviewed, including the *Great Encyclopedia of Chinese Medicinals* (中藥大辭典 *Zhong Yao Da Ci Dian*), the *Sea of Chinese Medicinals* (中華藥海 *Zhong Hua Yao Hai*), the *National Collection of Chinese Herbal Medicines* (全國中草藥彙編 *Quan Guo Zhong Cao Yao Hui Bian*), the *Chinese Materia Medica* (中華本草 *Zhong Hua Ben Cao*), and the *Chinese Great Encyclopedia* (中華大典 *Zhong Hua Da Dian*). Modern compilations focused on Chinese herbal formulas and ethnic minority medical traditions in Western China were also reviewed, including texts on Uighur, Yao, Miao, and Tibetan medicine. Additionally, publications from English and Chinese scientific journal databases such as CNKI, Wanfang, Google Scholar, and Scopus were analyzed based on a wide range of search terms related to Chinese medicine and cannabis in both English and Chinese.

## Approach to translation of technical terms

Preserving the traditional terminology of Chinese medicine is essential in order for translations to capture the original meaning of historical sources. In this work, a source-oriented approach to translation is utilized; in most cases, the technical terms used in translation can be cross referenced to the original Chinese using the terminology established in *A Practical Dictionary of Chinese Medicine* (Wiseman and Feng, 1998). Additional bilingual term lists referenced in the process of translation include: the *Dictionary of the Ben cao gang mu, Volume 1: Chinese Historical Illness Terminology* (Zhang and Unschuld, 2014), the *WHO International Standard Terminologies on Traditional Medicine in the Western Pacific Region*, and *International Standard Chinese-English: Basic Nomenclature of Chinese Medicine*.

In the context of cannabis, several unique challenges relate to the translation of the plant parts used, as historical sources offer conflicting descriptions of different plant parts. In particular, historical and contemporary sources are divided regarding the botanical structures that correspond to the terms *mafen* (麻蕡), *mahua* (麻花), and *mabo* (麻勃), as well as terms related to the fruit or seed such as *mazi* (麻子), *maziren* (麻子仁), and *mashi* (麻實). In this review, these terms are preserved via a combination of English, Pinyin, and Chinese, and the implications of their historical and contemporary confusion are discussed.

## Identification of traditional actions and indications that may reflect cannabinoids

In contemporary Chinese medicine, fiber-rich biotypes of cannabis provide the achenes used as *huomaren* (Cannabis Fructus). However, as the achenes of cannabis contain at most only trace amounts of cannabinoids such as CBD and Δ^9^-THC (Mölleken and Husmann, 1997), the indications of the achenes are unlikely to relate to cannabinoids and their observed effects are unreliable for differentiating the historical prevalence of different biotypes of cannabis in Chinese medical applications. Similarly, Chinese medical records related to other plant parts with minimal cannabinoid content, such as the stalks and roots of cannabis, are of limited value for differentiating historical biotypes or evaluating applications that may relate to cannabinoids. Thus, the primary plant parts that can be reasonably expected to illustrate effects that relate to cannabinoids are the leaf and female inflorescence. Accordingly, while the actions, indications, and properties of all parts of the cannabis plant were reviewed in the historical texts described above, the female inflorescence and leaf formed the focus of the investigation.

A review of the nature, flavor, actions, and indications of various cannabis plant parts in Chinese *bencao* reveals a number of terms that may indicate the presence of intermediate or drug biotypes of cannabis. For example, applications related to severe pain, perceived toxicity, or actions such as inducing anesthesia or hallucinations may reflect the historical presence of drug biotypes. Accordingly, the terminology associated with such effects was compared with descriptions of other drugs found in the Chinese materia medica that have known hallucinogenic or narcotic effects, such as *Datura* spp., *Hyoscyamus niger* L., and *Papaver somniferum* L. Additionally, the *bencao* literature was reviewed for other medical applications that have been associated with cannabinoid-rich preparations, including applications for conditions such as epilepsy and mental illness that may be related to the pharmacology of cannabidiol CBD rather than psychoactive cannabinoids such as Δ^9^-THC (Mechoulam et al., 2002; Devinsky et al., 2014).

## Results and discussion

Cannabis has been documented in *bencao* texts from the Eastern Han Dynasty (c. 200 AD) up through the twentieth century materia medica literature. All parts of the plant were recorded in *bencao* texts by 659 AD, but the inflorescence (*mafen*) and the “seed” (achene fruit) tend to appear more frequently as monograph headings than the leaf, root, and cortex (Zheng, 2008); the achenes are the only plant part that remains used in modern clinical practice. As described below, the *bencao* literature suggests that both drug and fiber biotypes were known in ancient China, but *bencao* texts never differentiated the plant into drug vs. fiber or oil varieties. Consequently, determining the implications of different biotypes on the historical applications of cannabis requires an in-depth analysis of the actions, indications, and plant parts used in ancient medical texts.

Furthermore, although records in the historical literature that suggest intoxication or altered consciousness may help to indicate preparations with significant levels of cannabinoids, such references may overlook effects that relate to non-psychoactive cannabinoids such as CBD, which are the predominant cannabinoids in the cannabis (hemp) varieties widely grown in China today. Consequently, indications cited in ancient texts for conditions such as epilepsy, seizures, and pain may in some cases relate to cannabinoids such as CBD rather than Δ^9^-THC (Mechoulam et al., 2002), making it difficult to reliably use overt drug effects as a proxy for identifying cannabinoid-related medical applications.

Our textual analysis suggests that drug biotypes of cannabis were known in ancient Chinese medicine, but it is possible that long-term selection of fiber-rich cultivars caused drug biotypes to fade in terms of their medical importance over time. Several trends in the literature suggest that drug biotypes of cannabis were rarely applied in Chinese medicine or gradually became less prominent, including: (1) the increased prominence of the achenes and reduced prominence of other plant parts such as the female inflorescence in the *bencao* literature over time; (2) enduring nomenclature confusion regarding plant parts, which suggests limited practical application and experience by later authors; and (3) actions and indications in many early texts that were repeated over the centuries but had relatively little elaboration and practical application in later *bencao* sources.

### Prominence of the achenes in clinical application

Many early *bencao* texts from the second century through the twelfth century AD feature the inflorescence (*mafen*) as a main monograph heading with the achenes (known as *mazi* or *maziren*) presented as an addendum, but over time the emphasis gradually tended to favor the achenes. For example, in the *Compendium of Materia Medica* (*Ben Cao Gang Mu*) from the late sixteenth century, ~3 times as many words are dedicated to the discussion of the properties and indications of the achenes in comparison with the inflorescence (Liu et al., 2009). By the time of the Qing Dynasty (1644–1911 AD), many *bencao* texts only contained monographs on the achenes, and the inflorescence (*mafen*) was often omitted entirely, a practice that has carried over into modern clinical textbooks of TCM.

Beyond the context of *bencao* texts, an emphasis on the use of the achenes in clinical practice can also be seen from TCM formula literature. For example, the achenes are featured as a key ingredient in the classical formula *Cannabis Seed Pill* (*Ma Zi Ren Wan* 麻子仁丸), which was first recorded by the physician Zhang Zhongjing around the second century AD and remains prominent in both clinical use and TCM textbooks in the modern era (Brand and Wiseman, 2008). According to data from the National Health Insurance system in Taiwan collected in 2003, this formula ranked #40 out of the 301 most frequently prescribed TCM formulas for insurance reimbursement, with over 10,705 kg of concentrated dry extract prescribed in Taiwan in 2003. In the same year in Taiwan, 967 kg of concentrated dry extract from the achenes (*huomaren*) was also prescribed for insurance reimbursement as a single-herb addition to formulas, ranking it as #140 of 353 single herb extracts by weight (Jian, 2006).

By contrast, the inflorescence of cannabis rarely appeared in historical formulas, with the exception of a relatively modest range of small formulas that are found in *bencao* texts. No named formulas that feature the inflorescence of cannabis are found in the *Great Encyclopedia of Chinese Medicinal Formulas* (中醫方劑大辭典 *Zhong Yi Fang Ji Da Ci Dian*), which contains 96,592 historical formulas and features 69 formulas that utilize the achenes of cannabis as a primary ingredient (Peng, 2005). While a small number of combinations with other herbs are listed in *bencao* texts, the relatively small number of compound formulas that feature the inflorescence of cannabis thus suggests that the inflorescence was rarely used in clinical applications when compared with the achenes.

Abundant *bencao* references to cannabis as a food and fiber crop suggest that fiber and seed production were emphasized from an early time. For example, in the *Collection of Commentaries on the Classic of the Materia Medica* (*Shen Nong Ben Cao Jing Ji Zhu*) from the sixth century AD, the author Tao Hongjing notes that cannabis was used to make cloth and shoes (Tao, 1999). Additional references to pressing the seeds for oil and using the fiber for cloth and candlewicks are also found in later texts such as the *Compendium of Materia Medica* (*Ben Cao Gang Mu*) by Li Shizhen in the sixteenth century (Liu et al., 2009). Furthermore, cannabis was frequently classified in *bencao* texts with other food crops under the heading of “grains,” which suggests that it had a prominent role as a food (Li, 2005). Hemp seed continues to be consumed as a food in modern China; it has a reputation as a “longevity” food in the Bama region of Guangxi province (Wang et al., 2010), and in Hong Kong beverages made of hemp seed are commonly sold in street stalls as well as in bottled products made by large companies including Coca-Cola.

However, despite the prominence of the achenes in ancient and modern applications, contemporary and historical texts contain contradictions related to nomenclature that remain poorly resolved. Although colloquially referred to as “seeds” in both English and Chinese, in botanical terms the brown, lustrous achenes with intact shells seen in TCM pharmacies today are fruits that contain seeds under the brown pericarp.

Confusion between the “fruit” vs. “seed” of cannabis is found in both ancient and modern sources. In the 2015 and 2005 editions of the Chinese Pharmacopeia (CP), *huomaren* (火麻仁) is defined as the dried mature fruit, resulting in the Latin drug name of “Cannabis Fructus” (CP, 2015). However, the name “Cannabis Semen” was featured in the 2010 edition of the Chinese Pharmacopeia, which stated its identity as the mature seed but featured a description of the fruit. In other contemporary texts such as the *National Collection of Chinese Herbal Medicines* (全國中草藥彙編 *Quan Guo Zhong Cao Yao Hui Bian*), the identity of *huomaren* is listed as either the fruit or the seed with the pericarp removed. By contrast, the *Chinese Materia Medica* (中華本草 *Zhong Hua Ben Cao*) and the *Great Encyclopedia of Chinese Medicinals* (中藥大辭典 *Zhong Yao Da Ci Dian*) list only the seed but feature macroscopic and microscopic descriptions that match the fruit (Editorial Committee, 1977; Zheng, 2008). Furthermore, in some historical texts it is difficult to definitively identity the botanical structures of cannabis that are referenced by traditional terms such as “fruit” (實 *shi*), “seed” (子 *zi*), “kernel/seed” (仁 *ren*), and “shell” (殼 *qiao*). This mirrors the challenges inherent in interpreting the complex terminology surrounding the terms used to refer to the female inflorescence (and infructescence) in *bencao* texts.

### Enduring confusion regarding plant parts: resolving traditional nomenclature

One of the most significant challenges for the interpretation of *bencao* records related to cannabis lies in the traditional terminology used to describe the flowering tops of the plant. Three terms are prominently used in *bencao* texts, and different authors from different historical eras describe them in contradictory ways. These terms, *mafen* (麻蕡), *mahua* (麻花), and *mabo* (麻勃), all refer to the spike-shaped inflorescence of the plant, but various contemporary and historical sources interpret them differently.

*Mafen* (麻蕡), which is the term most frequently encountered as a heading for cannabis in ancient *bencao* texts, is often defined as the immature inflorescence of the female flower or the mature infructescence of the seeded female flower (Liu and Shang, 1992; Liu, 1999). It was first listed in the *Divine Farmer's Classic of Materia Medica* (神農本草經 *Shen Nong Ben Cao Jing*, c. second century), wherein the term *mabo* (麻勃, literally “cannabis spike”) was listed as a synonym but no physical descriptions were provided. As the text was transmitted, commentary known as *Additional Records of Famous Physicians* (*Ming Yi Bie Lu*) was added to clarify that *mafen* was the “rising spike on the cannabis flower,” and should be harvested on the 7th day of the 7th month (based on the lunar calendar). This harvest time stands in contrast to the achenes, which were harvested in the 9th month according to the same source (which also noted that *mafen* is “toxic” while the achenes are non-toxic; Tang, 1999).

In the sixth century, the *Divine Farmer's Classic of Materia Medica* and the *Additional Records of Famous Physicians* were transmitted along with additional commentary from Tao Hongjing, a physician and Taoist alchemist who utilized different colors of ink to differentiate his annotations from the original transmitted text. Tao's comments initiated centuries of debate because he described *mafen* as cannabis without fruit, which clashed with previous definitions from the *Er Ya* dictionary that described *mafen* as the fruit of cannabis. Later authors disagreed about the identity of *mafen* (麻蕡), *mahua* (麻花 “cannabis flower”), and *mabo* (麻勃 “cannabis spike”), with some sources regarding the terms as synonymous while others regarded them as separate entities using poorly defined concepts of fruit vs. flower, leading later sources to divide them into multiple entries. Notably, Li Shizhen attempted to rectify these nomenclature issues in the sixteenth century while dividing *mabo* and *mafen* into separate monographs in the *Compendium of Materia Medica*, but his analysis introduced additional confusion that carried over into modern materia medica compilations and remains unresolved to this day.

The complex debate surrounding the nomenclature of *mafen* has been summarized in several contemporary Chinese journal publications (Liu and Shang, 1992; Liu, 1999). In addition to the immature female inflorescence and the mature seeded female infructescence, modern *bencao* scholars have also proposed that the identity of *mafen* includes the bracts surrounding the achenes but not the achenes themselves (Chen and Huang, 2005). Prominent historical *bencao* authors also discussed the meaning of *mafen*, including Su Jing (659 AD), Su Song (1062 AD), and Tang Shenwei (1108 AD; Zheng, 2008). The author Li Shizhen summarized the disparate views advanced by earlier historical authors in the *Compendium of Materia Medica* (*Ben Cao Gang Mu*) in 1593 AD, and Li's descriptions and corrections were influential in shaping the definitions established by contemporary texts such as the *Great Encyclopedia of Chinese Medicinals* (*Zhong Yao Da Ci Dian*).

In the *Compendium of Materia Medica*, Li summarized the discrepancies between the *Divine Farmer's Classic of Materia Medica*, Su Jing and Su Song's descriptions. Li chose to follow descriptions from *Wu Pu's Materia Medica* (*Wu Pu Ben Cao*) that established synonymy between the terms *mabo* (cannabis spike) and *mahua* (cannabis flower), which were classified as acrid and non-toxic, and separated these terms from *mafen*, which was classified as acrid, sweet, and toxic. Thus, despite the fact that the *Divine Farmer's Classic of Materia Medica* listed *mabo* as a synonym of *mafen*, Li Shizhen split the two into separate entries.

For the entry on *mabo*, Li listed *mahua* (cannabis flower) as a synonym, and cited a statement from the sixth century agricultural text *Essential Techniques for the Welfare of the People* (*Qi Min Yao Shu*) that referenced removing the males at the time that the plant produces “*bo*” (the budding spike-shaped flower) as evidence that “*bo* clearly refers to the flower” (Liu et al., 2009). Although it is unclear whether the term “*bo*” refers to the male or female flower in the text cited, Li's classification was later adopted by the *Great Encyclopedia of Chinese Medicinals*, which lists the male flower under the heading of *mahua* (Editorial Committee, 1977).

In the entry on *mafen*, Li stated that *mafen* refers to *mazi* (the achenes or possibly the mature infructescence) with the “shell” (*qiao*) intact (Liu et al., 2009). Li noted that *mafen* was different from edible cannabis because the shell is toxic and the inner kernel is non-toxic, but his description failed to adequately clarify whether the shell was a reference to the pericarp or the bract surrounding the achene fruit. In the contemporary *Great Encyclopedia of Chinese Medicinals*, the term *mafen* is defined as the immature racemes (Editorial Committee, 1977).

The question of which plant tissues correspond to *mabo, mahua*, and *mafen* is thus poorly resolved, as early *bencao* texts regarded them as synonyms but some later texts divide them by gender. For example, *mahua* is regarded as a synonym of *mabo* in the *Chinese Materia Medica* (*Zhong Hua Ben Cao*); this text describes it as the male flower but its pharmacology section refers only to Δ^9^-THC, which is not found in significant quantities in the male flower. In the *Sea of Chinese Medicinals* (*Zhong Hua Yao Hai*), the term *mabo* is listed as a synonym for *mafen*, which is described as the immature racemes (Cui and Ran, 1993). The sixth century author Tao Hongjing listed *mabo* as a synonym of *mafen* and stated that *mafen* lacked fruit (Tao, 1999), which along with the non-toxic properties ascribed to *mabo* in later texts may have influenced its classification as the male flower; however, no major Chinese texts have proposed that *mafen* lacking fruit could refer to the seedless female inflorescence.

The wide range of different interpretations for the identity of *mafen* presented over the centuries suggests that many later authors were preserving previous quotations yet had little practical experience with materials such as *mafen, mabo*, and *mahua*. Indeed, the author Tao Hongjing noted as early as the sixth century AD that “*mafen* was rarely used in formulas” (Tao, 1999).

### Applications of cannabis in the Chinese medical literature that may relate to cannabinoids

The earliest historical references to cannabis in Chinese medicine are found in the *Divine Farmer's Classic of Materia Medica* (*Shen Nong Ben Cao Jing*) from the first to second century AD. This text, along with the added notes known as the *Additional Records of Famous Physicians* (*Ming Yi Bie Lu*), contains many of the fundamental statements that were repeated about cannabis in later centuries.

The original text of the *Divine Farmer's Classic of Materia Medica* ascribes the following properties to *mafen:* “Flavor: acrid; balanced. Governs the five taxations and seven damages, benefits the five viscera, and descends blood and cold qi; excessive consumption causes one to see ghosts and run about frenetically. Prolonged consumption frees the spirit light and lightens the body. Another name is *mabo*” (Tao, 1999). To this base description, the *Additional Records of Famous Physicians* adds that it is “toxic,” and is used to “break accumulations, relieve impediment, and disperse pus” (Liu et al., 2009).

Many authors have interpreted the statement “excessive consumption [of *mafen*] causes one to see ghosts and run about frenetically” as evidence that *mafen* had drug effects due to the presence of cannabinoids such as Δ^9^-THC (Chen and Huang, 2005). Additionally, the reference to “relieving impediment” from the *Additional Records of Famous Physicians* refers to a traditional category of conditions that typically result in pain and restricted movement, which may also relate to cannabinoids such as CBD or Δ^9^-THC.

These original statements were repeated in many later *bencao* texts, and have likely influenced the properties listed for *mafen* in contemporary Chinese texts. For example, the *Great Encyclopedia of Chinese Medicinals* states that *mafen* “dispels wind, relieves pain, and settles tetany” (a traditional disease category associated with severe spasm). According to this text, it is indicated for conditions traditionally known as “impediment patterns” (typically manifesting in pain and restricted movement), gout, withdrawal and mania, insomnia, and panting and cough (Editorial Committee, 1977). Additionally, in the 1935 text *Illustrated Analysis of Medicinal Substances* (*Yao Wu Tu Kao*), the ancient statement that cannabis descends blood and cold qi was interpreted by the author Yang Huating as an indication that *mafen* quickens the blood. Yang recommended *mafen* (which he regarded as the female inflorescence) for a variety of conditions including headache, menstrual irregularities, itching, convulsions, anemia, and dry cough (Editorial Committee, 1977). However, despite these twentieth century publications that summarize traditional indications using contemporary descriptions, only a few texts offered new information or applications for *mafen* between the sixteenth century and Yang's 1935 text (Zheng, 2008).

#### Applications for pain

Several applications of cannabis for pain in Chinese medicine may relate to cannabinoids. As noted above, the first reference to *mafen* for pain is found in the *Additional Records of Famous Physicians* from the sixth century AD, which notes its use for “relieving impediment” (impediment is a traditional disease category that is also known as “*bi*” or “painful obstruction”; Wiseman and Feng, 1998). Some authors also speculate that an early anesthetic formula known as “*mafei powder*” (*ma fei san*) developed by the famous physician Hua Tuo around the turn of the third century AD contained *mafen* (at the time, the name *mafen* is believed to have shared the same pronunciation as the characters in the formula name; Chen and Huang, 2005). However, any link between cannabis and Hua Tuo's formula is purely speculative, as the original ingredients of the ancient formula *ma fei san* are lost.

In another application related to pain, the Tang Dynasty physician Sun Simiao (581–683 AD) recorded that the leaves of cannabis could be crushed to extract their juice, which was used to treat unbearable pain due to fractured bones (Chen and Huang, 2005).

By 1070 AD, the Song Dynasty text *Illustrated Classic of Materia Medica* (*Tu Jing Ben Cao*) included a quotation from a previous source titled *Formulas Within a Small Box* (*Qie Zhong Fang*) that referenced a preparation of cannabis for severe pain that inhibited movement (Su, 1994). In the original recipe, the preparation method specifies that the seeds of cannabis are soaked in water, then the sediment is collected from the bottom of the water, stir-fried until aromatic in a silver vessel, and ground into a fine white powder; this is then boiled with alcohol and taken internally on an empty stomach (Su, 1994). It is indicated for “bone marrow wind toxin” with pain that prevents movement; the text says that even in severe cases, “by 10 servings the suffering must be alleviated; its effect cannot be surpassed” (Zheng, 2008). This prescription was repeated in many later texts under the name “cannabis seed wine” (*da ma ren jiu*) under entries for the achenes (Editorial Committee, 1977); however it differs strikingly from other preparations of the achenes because it is used for severe pain. If the achenes were soaked in water with the bracts intact, it is possible that the preparation method described would yield cannabinoids, as broken resin glands from the bracts would sink in water; when this sediment was stir-fried, THC acids would be decarboxylated into bioavailable THC, which would then be efficiently extracted when boiled with alcohol, as in the original preparation. Nonetheless, while cannabinoids offer a plausible explanation for the unusual effects and preparation methods used for this formula, such an interpretation remains purely speculative in the absence of confirming evidence.

The first well-documented application of cannabis in an anesthetic formula in China appeared in the text *Heart Text of Bian Que* (*Bian Que Xin Shu*, 1127–1270 AD). The flower of cannabis (under the name *mahua*) was used internally in combination with datura flower (*Datura* spp.) as an anesthetic to decrease the sensation of pain when moxa cones were applied (Dou, 1992). This remedy was known as “sagacious sleep powder” (*shui sheng san*). The source text notes that it induces a stupor-slumber in which the person experiences no pain and is not harmed. The same combination is repeated in the *Compendium of Materia Medica* in the sixteenth century, which contains an additional recipe for wind disease with numbness that combines cannabis flower (*mahua*) with wild aconite root (*caowu*; Liu et al., 2009). By the seventeenth century, the text *Reaching the Source of Materia Medica* (*Ben Jing Feng Yuan*) reported that cannabis flower (*mahua*) can treat hidden wind within the body, and records further that it is used as an anesthetic, noting that it can be used to painlessly apply a stone needle to swollen welling-abscesses (Zhang, 2011).

#### Applications that relate to mental effects or mental illness

A variety of historical sources describe mental effects from cannabis or applications to treat mental illness. In some cases, these applications may reflect cannabinoids, as CBD has been researched for anti-psychotic effects (Mechoulam et al., 2002) and some of the mental effects described may be related to the effects of cannabinoids such as Δ^9^-THC. As noted above, the early statement that “excessive consumption causes one to see ghosts and run about frenetically” is often regarded as a sign of mental effects (Liu and Shang, 1992). In the sixteenth century *Compendium of Materia Medica*, Li Shizhen repeated this statement and added a previous recipe that states: “for those seeking to see ghosts, take unprocessed cannabis [the text says “cannabis seeds” (*sheng ma zi*) but lists the recipe under the entry for *mafen*], acorus rhizome (*shi chang pu, Acorus* spp.) and dysosma (*gui jiu, Dysosma* spp.) in equal parts and form into pellet pills. Take one pill every morning facing the sun and after 100 days one will see ghosts” (Liu et al., 2009). Additionally, in 973 AD, the *Materia Medica of the Kaibao Era* (*Kai Bao Ben Cao*) quoted an earlier author from the eighth century with the statement that “cannabis causes happiness in the heart” (Zheng, 2008).

Other early quotations suggest that mental effects were observed from the use of cannabis. For example, in the sixth century *Collection of Commentaries on the Classic of the Materia Medica* (*Ben Cao Jing Ji Zhu*), the author Tao Hongjing noted: “adepts (likely referring to Taoist alchemists) take cannabis flower (*mabo*) with ginseng and know of things that have not yet come” (Tang, 1999). In the *Compendium of Materia Medica* in the sixteenth century, the author Li Shizhen regarded this as “an overstatement,” instead stating that the combination of ginseng and cannabis allows one to “know the affairs of the four directions” and treats forgetfulness. The *Compendium of Materia Medica* also noted that the leaf of cannabis was indicated to treat malaria and was said to induce a state of drunkenness (Liu et al., 2009).

In addition to mental effects observed from the use of cannabis, historical *bencao* texts featured applications for cannabis in the context of mental illness. The first appearance of these applications dates to the seventh century text *Formulas Worth a Thousand Gold* (*Qian Jin Fang*) by Sun Simiao (Tang, 1999), which stated that cannabis treated wind-withdrawal, a traditional category of disease that relates to mental illness (Wiseman and Feng, 1998). Similar indications are ascribed to the same quote from Sun Simiao by Li Shizhen in the *Compendium of Materia Medica*, which lists wind-withdrawal and “the 100 diseases” as indications for *mafen*. By the Qing Dynasty, the seventeenth century text *Reaching the Source of Materia Medica* (*Ben Jing Feng Yuan*) stated that cannabis flower (*mahua*) treats “120 types of malign wind” as well as itching, and expels all malign wind and blood; it was also indicated to treat lack of free flow following menstruation. These quotations may have inspired the actions listed for *mafen* in twentieth century texts such as “dispelling wind” and treating mania-withdrawal (a traditional category of mental illness in Chinese medicine).

In some cases, it is possible that actions that were ascribed to *mafen* in twentieth century Chinese texts were acquired from Western applications of cannabis. For example, the 1905 text *Pharmacognosy* (*Sheng Yao Xue*) by Li Chenghu stated that cannabis treated agitation, hysteria, spasmodic cough, and nerve pain, while the 1935 text *Illustrated Analysis of Medicines* (*Yao Wu Tu Kao*) by Yang Huating added many new indications such as mania-withdrawal, convulsions, and insomnia that were not previously discussed in historical texts (Editorial Committee, 1977).

In the case of traditional actions seen in contemporary Chinese medical texts for *mafen* such as settling tetany (a traditional disease term associated with severe spasm and convulsions; Editorial Committee, 1977), it remains unclear whether this action is related to assimilation of Western medical theories in the early twentieth century or whether it is derived from traditional indications for wind and wind-withdrawal. In ancient times, the line between “withdrawal” (*dian*) as a mental disease sometimes overlapped with the concept of epilepsy (*dian xian*), which is characterized by seizures and is associated with “wind” in traditional Chinese medical theory (Wiseman and Feng, 1998). However, the historical record provides insufficient detail to ascertain whether the seventh century indications for cannabis in the context of wind-withdrawal overlapped with epilepsy or seizures, or whether the meaning was primarily limited to mental illness and/or erratic behavior.

## Conclusion

In recent years, cannabinoids such as CBD and Δ^9^-THC have attracted increased attention in the context of modern pharmacology and popular Western culture, yet little research has been done to explore the historical applications of cannabis in Chinese medicine. Given China's long history of hemp cultivation and its rich body of un-translated medical literature, it is surprising that little academic attention has focused on exploring the ways in which cannabis was used in Chinese medicine. The importance of cannabis as a fiber and food crop in ancient China, combined with the extensive use of the achenes in medicine, makes the Chinese historical record particularly valuable.

*Bencao* literature opens a window into the history and culture of ancient Chinese medicine. As all parts of the cannabis plant were recorded in *bencao* texts, the Chinese medical literature can help to clarify many details about the historical applications of cannabis in Chinese medicine, as well as providing clues into the historical prevalence of different biotypes as ancient Chinese farmers gradually selected superior varieties for fiber and seed crops. The significant differences in how cannabis has been employed in Chinese vs. Western medicine likely relate to differences between drug and fiber biotypes as well as cultural factors, but thus far minimal research has focused on exploring this issue. Similarly, minimal attention has been given to the topic of CBD in Chinese medical history, as even fiber-rich biotypes of cannabis that were not associated with drug use may have had potential therapeutic applications related to CBD. While this modest review can only scratch the surface of the Chinese medical literature of cannabis and the questions it raises, it is hoped that further research will help to further elucidate these questions using a multidisciplinary approach.

## Author contributions

EB: Primary research and manuscript creation. ZZ: Expert review, source suggestions, revisions, and feedback.

### Conflict of interest statement

The authors declare that the research was conducted in the absence of any commercial or financial relationships that could be construed as a potential conflict of interest.
